# The Emerging Role of m6A and Programmed Cell Death in Cardiovascular Diseases

**DOI:** 10.3390/biom15020247

**Published:** 2025-02-08

**Authors:** Haixia Wang, Juanjuan Han, Hui Kong, Ce Ma, Xin-an Zhang

**Affiliations:** 1 College of Exercise and Health, Shenyang Sport University, Shenyang 110102, China; whx1415@163.com (H.W.); hanhan9210@163.com (J.H.); 17852060669@163.com (H.K.); 2 College of Exercise and Health, Shanghai Sport University, Shanghai 200438, China; 3 Sports Training Teaching and Research Office, Shenyang Sport University, Shenyang 110102, China; mace_5055@163.com

**Keywords:** N6-methyladenosine (m6A), programmed cell death, myocardial ischemia, pulmonary hypertension, atherosclerosis, exercise

## Abstract

N6-methyladenosine (m6A) is the most prevalent internal chemical modification in eukaryotic messenger RNA (mRNA), significantly impacting its lifecycle through dynamic and reversible processes involving methyltransferase, demethylase, and binding proteins. These processes regulate mRNA stability, splicing, nuclear export, translation, and degradation. Programmed cell death (PCD), a tightly controlled process encompassing apoptosis, pyroptosis, ferroptosis, autophagy, and necroptosis, plays a crucial role in maintaining cellular homeostasis, tissue development, and function. Recently, m6A modification has emerged as a significant research area due to its role in regulating PCD and its implications in cardiovascular diseases (CVDs). In this review, we delve into the intricate relationship between various PCD types and m6A modification, emphasizing their pivotal roles in the initiation and progression of CVDs such as myocardial ischemia-reperfusion (I/R), atherosclerosis (AS), pulmonary hypertension (PH), cardiomyopathy, doxorubicin (Dox)-induced cardiotoxicity (DIC), heart failure (HF), and myocardial infarction (MI). Our findings underscore the potential of elucidating the roles of m6A and PCD in CVD to pave new pathways for prevention and treatment strategies.

## 1. Introduction

The most prevalent kind of internal RNA modification in eukaryotes is called N6-methyladenosine (m6A), which is found in both mRNA and non-coding RNA (ncRNA) [1,2]. Unlike other mRNA modifications, m6A modification is dynamic and reversible, and it is mediated by three different types of regulatory factors: “erasers” (demethylation executors like FTO, ALKBH3/5, etc.), “writers” (methylation executors like METTL3, METTL14, etc.), and “readers” (m6A site recognizers like YTH family, IGF2BPs, etc.) [3,4]. This alteration may have an impact on RNA metabolism, which might contribute to post-transcriptional regulation and protein expression [5]. There is evidence to suggest that m6A can regulate the expression and function of programmed cell death (PCD), including apoptosis, pyroptosis, ferroptosis, autophagy, and necroptosis [6]. However, the close association between m6A and PCD is a key factor affecting the bio-logical processes of various diseases.

PCD denotes an active cellular demise process triggered by specific signals or stimuli to uphold internal environmental stability. It encompasses diverse forms such as classical apoptosis, pyroptosis, autophagy, necroptosis, and ferroptosis, most of which involve disruption of the plasma membrane [7,8]. PCD plays a pivotal role in sustaining normal organismal development, tissue homeostasis, and the elimination of damaged or infected cells [9,10]. More and more evidences show that PCD process is regulated by m6A modification related factors and plays an important role in the development of cardiovascular dis-eases (CVD) [11-13].

CVD is a kind of disease involving the heart and vascular system, causing ischemic or hemorrhagic lesions in body tissues, primarily impacting myocardial cells, vascular endothelial cells, and vascular smooth muscle cells. It encompasses conditions such as myocardial ischemia-reperfusion (I/R), atherosclerosis (AS), pulmonary hypertension (PH), cardiomyopathy, doxorubicin (Dox)-induced cardiotoxicity (DIC), heart failure (HF), and myocardial infarction (MI) [14-17]. In recent years, the incidence of CVD has continued to rise, becoming a major contributor to global disability and posing substantial pressure on global health and social security systems [18]. However, the precise underlying mechanisms remain elusive, and effective treatment options and medications are currently lacking [19,20]. Accumulating evidence suggests that m6A modification is intimately linked to CVD and is recognized as a crucial regulatory factor involved in modulating PCD [21,22]. In this review, we underscore the functional interactions between m6A regulatory factors and various PCD types and further elucidate the pertinent molecular mechanisms underlying the roles of m6A and PCD in CVD.

## 2. M6A Modification

**Figure 1:**
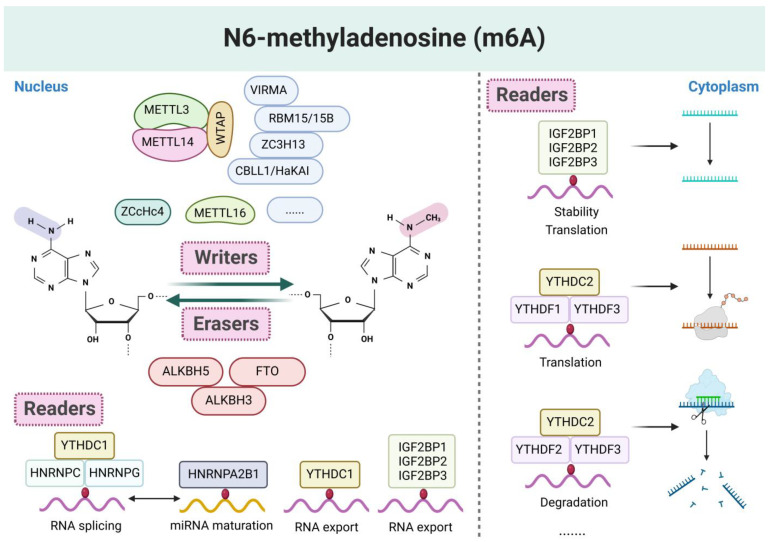
The makeup and process of methylation of m6A RNA. Three molecular components are involved: m6A recognition factor (readers), m6A demethylase (erasers), and m6A methyltransferase (writers). Take part in controlling a number of intricate biological processes involving RNA, including splicing, processing, translation, and destruction.

M6A represents the methylation modification occurring on the sixth nitrogen atom of adenine within RNA molecules. It stands as the most prevalent post-transcriptional modification in messenger RNA (mRNA) and ncRNA [23]. Through meticulous regulation of precursor mRNA splicing, stability, degradation, translation efficiency, mRNA decay, and nuclear-cytoplasmic transport, m6A modification profoundly influences RNA expression patterns and functions, extensively engaged in the regulatory network of ncRNA [24,25]. This dynamic and reversible modification encompasses three fundamental steps: “writing”, “erasing”, and “reading” [26]. These three components collectively orchestrate alterations in the m6A loci, playing a vital role in sustaining normal biological functions and developmental processes of human tissues and cells. The “writing” process of m6A modification is orchestrated by m6A methyltransferases (Writers), primarily led by methyltransferase-like 3 (METTL3), with supplementary proteins like methyltransferase-like 14/5 (METTL14/5) and Wilm’s tumor-associated protein (WTAP). METTL3 functions as the catalytic core, employing its S-adenosylmethionine (SAM) binding domain and DPPW (Asp-Pro-Pro-Trp) activation motif to facilitate the transfer of methyl groups from SAM to the 6th nitrogen atom of adenine in RNA [27]. While METTL14 lacks direct methyltransferase activity, its degraded SAM binding domain and EPPL (Glu-Pro-Pro-Leu) motif are pivotal for stabilizing the METTL3-METTL14 heterodimer complex and amplifying METTL3’s catalytic activity [28]. WTAP, devoid of methyltransferase activity, facilitates the nuclear plaque localization of the METTL3-14 complex, ensuring precise methylation reactions [29]. Auxiliary proteins such as METTL16, KIAA1429 (alias VIRMA), zinc finger CCCH-type contain ning 13 (ZC3H13), RNA-binding motif protein 15/15B (RBM15/15B), CCHC zinc-finger-containing protein ZCCHC4, and NOL1/NOP2/Sun domain family member 2 (NSUN2) direct the methyltransferase complexes to specific RNA targets, thereby enhancing m6A modification. The “erasing” process is executed by demethylases (erasers), primarily fat mass and obesity-associated protein (FTO) and alkB homologue 5 (ALKBH5) enzymes. FTO catalyzes the oxidation of m6A to intermediate products, which are subsequently hydrolyzed back to adenine. ALKBH5 directly reverses the m6A modification. Both enzymes necessitate α-ketoglutarate (α-KG) and Fe^2+^ as co-substrates for their demethylation activities [30,31]. The “reading” process involves m6A reader proteins (readers), which specifically recognize and bind to m6A-modified RNA, thereby modulating its structure and function. Notable readers include the YTH domain family (YTHDF1/2/3, YTHDC1/2), insulin-like growth factor-2 mRNA-binding proteins (IGF2BP1/2/3), heterogeneous nuclear ribonucleoprotein (HNRNPA2B1, HNRNPC, HNRNPG), eukaryotic translation initiation factor 3 (eIF3), and rich pentatricopeptide repeat-containing protein (LRPPRC). Among them, YTHDF2 is extensively studied for its role in promoting m6A-dependent RNA degradation, while YTHDF1 and YTHDF3 may augment RNA translation efficiency [32,33]. Additionally, the IGF2BP proteins, another class of m6A readers, interact with m6A-modified RNA through their RNA binding domain, safeguarding mRNA from degradation and promoting translation and transport processes [34] (Figure 1). 

## 3. M6A and Programmed Cell Death

PCD, which comprises a variety of processes such as apoptosis, pyroptosis, ferroptosis, autophagy, and necroptosis, is a crucial mechanism for controlling cell death [35]. It is essential for the proper growth and upkeep of cellular tissue homeostasis and includes the activation, expression, and control of many genes. The sophisticated and multilayered regulation mechanism of PCD involves changes to cell shape, molecular interactions, and many levels of signal transduction. Moreover, epigenetics also controls the regulation of PCD [36]. The ability of m6A alteration to mediate PCD, which is linked to apoptosis, pyroptosis, ferroptosis, autophagy, and necroptosis and influences the development of several human illnesses, has been demonstrated in an increasing number of studies [37]. Undoubtedly, the association between m6A and PCD pathways will bring new breakthroughs and insights for the management and treatment of related diseases.

### 3.1. M6A and Apoptosis

**Figure 2:**
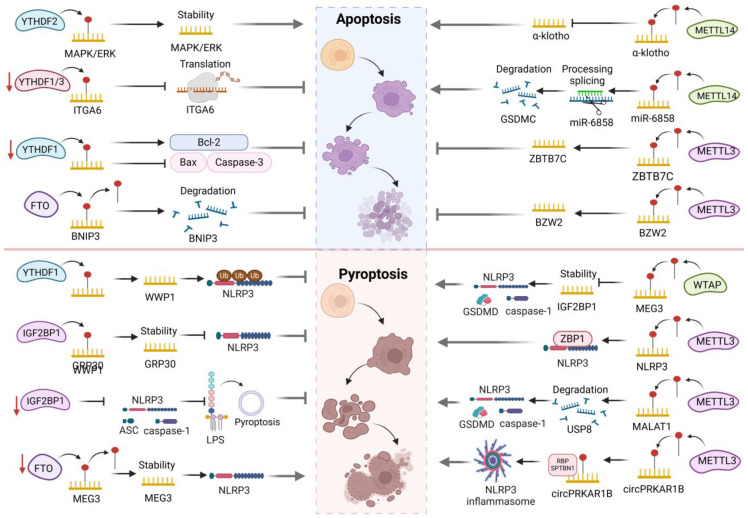
The mechanism of m6A regulating cell apoptosis and pyroptosis. In apoptosis, reader (YTHDF2) and writer (METTL14) act on related genes, promoting apoptosis by affecting the stability, activity, and degradation of RNA. The eraser (FTO) and writer (METTL3) inhibit apoptosis by regulating the expression and stability of related RNAs. The deletion of readers (YTHDF1/3) reduces apoptosis by inhibiting RNA translation. In pyroptosis, readers (YTHDF1 and IGF2BP1) act on related genes and exert inhibitory effects on pyroptosis by affecting RNA stability. The writers (WTAP and METTL3) promote pyroptosis by regulating the stability and degradation of related RNAs. The absence of eraser (FTO) promotes pyroptosis by affecting RNA stability.

M6A modification controls the stability, transport, and translation efficiency of mRNA, which in turn regulates the expression of genes linked to cell death. In the context of cancer, inhibition of YTHDF2-mediated mRNA degradation can induce apoptosis of triple-negative breast cancer (TNBC) cells and tumors[38]. In fact, YTHDF2 interacts with mRNA in the MAPK/ERK pathway, which stabilizes and promotes upregulation of epithelial mesenchymal transition (EMT) markers, translation surges, endoplasmic reticulum (ER) stress, and cell apoptosis [38]. In contrast, the absence of YTHDF1 weakens the ability of RNA-binding proteins to recognize m6A, hinders mRNA translation, and affects downstream biological functions. For example, in the transcription of integrin subunit alpha 6 (ITGA6), m6A exhibits a highly enriched active state, while knockout of YTHDF1 and YTHDF3 may hinder the translation of ITGA6 messenger RNA through the cell adhesion molecule signaling pathway, thereby reducing neuroinflammation and cell apoptosis [39]. Similarly, YTHDF1-knockout mice inhibit traumatic brain injury-induced cell apoptosis, inflammation, and edema, leading to increased levels of anti-apoptotic b-cell lymphoma 2 (Bcl-2) protein and decreased levels of pro-apoptotic proteins (BAX, caspase-3, and cleaved caspase-3), thereby alleviating brain–gut axis dysfunction in the mouse [40]. Different from YTHDF2, the high expression of FTO significantly promotes the proliferation, invasion, and migration of breast cancer cells and reduces apoptosis [41]. BCL2/adenovirus E1B interacting protein 3 (BNIP3), as a tumor suppressor, can activate caspase-3 and inhibit Bcl-2 expression. FTO can reduce apoptosis of breast cancer cells and promote their growth by demethylating m6A in the 3′UTR of BNIP3 mRNA and inducing its degradation in a YTHDF2-independent manner [42]. In addition, the highly expressed METTL14 downregulates the expression of α-klotho in an m6A-dependent manner, promoting inflammation, oxidative stress, and apoptosis in human renal glomerular endothelial cells [43]. Overexpression of METTL14 also plays a role in promoting apoptosis in acute kidney injury and renal I/R injury [44,45]. It is worth noting that NF-κB activation induces upregulation of METTL14 expression, thereby increasing m6A levels in the upper cortex of oral lichen planus. The m6A modification facilitates the processing and splicing of miR-6858, ultimately resulting in an augmentation of miR-6858 levels. This, in turn, leads to a heightened degradation of Gasdermin C (GSDMC) mRNA, ultimately triggering apoptosis in oral mucosal keratinocytes [46]. An increase in METTL3 levels accelerates the progression of both multiple myeloma and osteosarcoma. On the one hand, METTL3 achieves this by inhibiting apoptosis and promoting OS progression through the creation of ZBTB7C m6A modification [47]. On the other hand, it enhances the expression of basic leucine zipper and W2 domains 2 (BZW2) by modulating its m6A modification, thereby accelerating MM cell proliferation and inhibiting apoptosis [48]. Furthermore, inhibiting METTL3 has been found to mitigate lipopolysaccharide (LPS)-induced apoptosis in mouse lung epithelial cells (MLE-12) [49]. In summary, m6A modification exerts an influence on the apoptotic state of cells by modulating the transcription and translation of apoptosis-related genes, silencing methylation or demethylase genes, and impacting apoptotic signaling pathways (Figure 2). 

### 3.2. M6A and Pyroptosis

Cellular pyroptosis is an inflammation-related and caspase-1 dependent PCD process that plays a crucial role in various diseases along with m6A [50]. Partial m6A modification plays a regulatory role in cell sensitivity to pyroptosis by influencing the dynamic alterations of NOD-like receptor thermal protein domain-associated protein 3 (NLRP3) inflammasomes. For instance, YTHDF1 can upregulate WW domain-containing ubiquitin E3 ligase 1 (WWP1), thereby promoting NLRP3 ubiquitination and inhibiting the activation of NLRP3 inflammasomes. Furthermore, YTHDF1 hinders caspase-1-mediated cleavage of GSDMD [51]. Secondly, the interaction between IGF2BP1 and extracellular vesicles derived from neural stem cells carrying YBX1 (NSC-derived EVs) increases the stability of m6A-modified G protein-coupled receptor 30 (GPR30). The upregulation of GPR30 further inhibits the activation of NLRP3 inflammasomes and neuronal pyroptosis, ultimately alleviating ischemic stroke [52]. Additionally, in IGF2BP1 knockout HK2 cells, NLRP3, ASC, and caspase-1 p20 are all downregulated, leading to reduced LPS-stimulated pyroptosis of renal tubular cells [53]. Undoubtedly, m6A can also play a role in promoting cell pyroptosis. The downregulation of FTO regulates the stability of lncRNA MEG3 in an m6A-dependent manner, thereby modulating the NLRP3/caspase-1/GSDMD signaling axis to activate neuronal cell pyroptosis [54]. In diabetes nephropathy and high glucose (HG)-treated HK-2 cells, WTAP is abnormally overexpressed, promoting the m6A methylation of NLRP3 mRNA and triggering the reduction in the stability of IGF2BP1. Subsequently, NLRP3 inflammasomes are activated, inducing cell death and upregulating inflammatory-related factors such as NLRP3, pro-caspase-1, active caspase-1, GSDMD, and GSDMD-N [55]. Conversely, knocking down WTAP also attenuates cell pyroptosis in rheumatoid arthritis fibroblast-like synoviocytes treated with TNF-α [56]. Furthermore, increased m6A methylation on NLRP3 mRNA mediated by METTL3 can enhance the interaction between Z-DNA-binding protein 1 (ZBP1) and NLRP3 proteins, promoting trophoblast cell apoptosis and leading to miscarriage [57]. In liver fibrosis models and activated Kupffer cells (KCs), the elevated expression of METTL3 enhances metastasis-associated lung adenocarcinoma transcript 1 (MALAT1) levels via m6A methylation and facilitates the degradation of ubiquitin-specific peptidase 8 (USP8) mRNA, subsequently reduces transforming growth factor β-activated kinase 1 (TAK1) regulation, enhances cell pyroptosis markers (NLRP3, caspase-1, GSDMD-N) and NF-κB p-p65 levels, thereby promoting macrophage pyroptosis and inflammation [58]. Of course, silencing METTL3 also reduces KCs pyroptosis and inflammatory response in the liver of alcoholic steatohepatitis (ASH) mice, potentially by affecting the splicing of pri-miR-34A [59]. Moreover, METTL3 partially mediates the m6A modification of circPRKAR1B, promoting its abnormal overexpression and interacting with the RNA-binding protein SPTBN1. This interaction induces the upregulation of caspase-1, IL-1β, NLRP3, ASC, and cleaved N-terminal GSDMD expression, activating NLRP3 inflammasome-mediated cell pyroptosis and impairing autophagy, ultimately exacerbating Crohn’s disease (CD) [60]. Overall, the interaction between m6A and cell pyroptosis may have significant pathological implications in various diseases (Figure 2).

### 3.3. M6A and Ferroptosis

**Figure 3:**
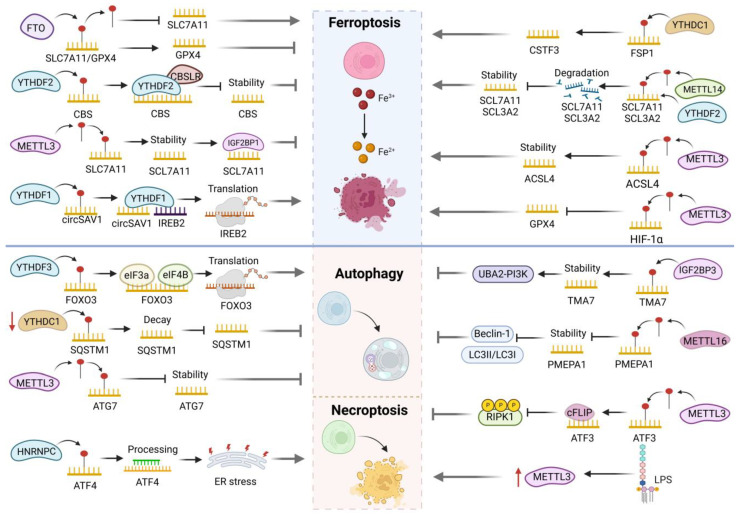
The mechanism of m6A regulating cell ferroptosis, autophagy, and necroptosis. In ferroptosis, eraser (FTO), readers (YTHDF1/2 and YTHDC1), and writers (METTL3/14) act on related genes, promoting ferroptosis by affecting the activity, stability, translation, and degradation of RNA. The eraser (FTO), reader (YTHDF2), and writer (METTL3) inhibit ferroptosis by regulating the expression and stability of related RNAs. In autophagy, reader (YTHDF3) promotes autophagy by affecting RNA translation. The writers (METTL3/16 and IGF2BP3) act on related genes and exert inhibitory effects on autophagy by affecting RNA stability. The deletion of reader (YTHDC1) inhibits autophagy by affecting RNA decay. In necroptosis, reader (HNRNPC) promotes necroptosis by affecting RNA processing. The writer (METTL3) promotes or inhibits necroptosis by regulating RNA activity.

A distinct method of cell death called ferroptosis is triggered by phospholipid peroxidation that is dependent on iron [61]. M6A modification can affect disease progression by regulating the expression of ferroptosis related genes (such as SLC7A11, GPX4, etc.). High expression of the ferroptosis related gene solute carrier family 7 member 11 (SLC7A11) inhibits cell death through ferroptosis. The tumor suppressor gene FTO reduces the progression of papillary thyroid cancer (PTC) by downregulating SLC7A11 [62]. Additionally, FTO knockout decreases glutathione peroxidase 4 (GPX4) expression in colorectal cancer (CRC) cells, promoting ferroptosis by modulating m6A modification at the 193rd site of GPX4 mRNA [63]. Similarly, when YTHDF2 forms a complex with CBSLR/YTHDF2/CBS, it destabilizes CBS mRNA in an m6A-dependent manner, leading to ACSL4 degradation via the ubiquitin-proteasome pathway and inhibiting ferroptosis in the hypoxic tumor microenvironment [64]. Mechanistically, METTL3-mediated m6A modification enhances SLC7A11 mRNA stability, while IGF2BP1 prevents CCR4-NOT-mediated deadenylation in an m6A-dependent manner, promoting ferroptosis resistance in hepatoblastoma cells [65]. Moreover, certain m6A-modifying enzymes promote ferroptosis. For instance, in lung epithelial cells, m6A facilitates YTHDF1 recruitment to circSAV1, allowing it to bind to the 5′UTR of iron-regulatory protein IREB2 mRNA, forming a circSAV1/YTHDF1/IREB2 ternary complex. This complex promotes the translation of IREB2 mRNA and induces ferroptosis in lung epithelial cells [66]. Furthermore, as an inhibitor of ferroptosis, FSP1 binds to YTHDC1 at the m6A site in its 3′UTR, recruiting CSTF3 to generate short, unstable FSP1 mRNA transcripts. Downregulation of YTHDC1 increases FSP1 protein levels, leading to ferroptosis resistance in lung cancer cells [67]. Conversely, upregulation of METTL14 accelerates the degradation of SLC7A11 and SLC3A2 mRNA via the m6A YTHDF2-dependent pathway, reducing their stability. This sequence of events triggers the accumulation of reactive oxygen species (ROS), inducing ferroptosis in CRC cells [68]. Additionally, the lactate-METTL3-YTHDC1-ASCL4 axis regulates mitochondrial-dependent ferroptosis. In vitro, lactate stimulation upregulates METTL3 expression by promoting p300-mediated binding of H3K18la to the METTL3 promoter, thereby enhancing the stability and half-life of ACSL4 and promoting iron-mediated cell death [69]. METTL3 transcription is also regulated by p300-catalyzed H3K27ac, which further enhances HIF-1α expression through an m6A IGF2BP2-dependent mechanism, thereby promoting ferroptosis in sepsis-induced acute lung injury by downregulating GPX4 [70]. In conclusion, ongoing research continues to uncover the intricate interactions between m6A modification and ferroptosis, potentially providing more precise and effective therapeutic strategies for related diseases (Figure 3). 

### 3.4. M6A and Other Programmed Cell Death

Autophagy and necroptosis are also influenced by m6A modification [71]. By regulating the expression of autophagy-related genes, m6A modification can impact the initiation and progression of autophagy. Recently, convincing evidence has shown that YTHDF3 promotes forkhead box O3 (FOXO3) translation and initiates autophagy by recognizing m6A modification sites near the FOXO3 mRNA termination codon and recruiting eIF3a and eIF4B subunits to its 5′ end [72]. Similarly, YTHDC1 deficiency inhibits autophagy in keratinocytes by accelerating SQSTM1 mRNA decay and reducing SQSTM1 expression, while its overexpression restores autophagy blocked by high glucose [73]. However, excessive m6A modification negatively regulates autophagy in osteoarthritis fibroblast-like synovial cells, as METTL3-mediated m6A reduces the stability of autophagy-related (ATG) 7 mRNA [74]. Knocking down METTL16 enhances autophagy by increasing Beclin-1 and LC3II/LC3I levels and decreasing p62. Further research suggests METTL16 inhibits bladder cancer cell proliferation and cisplatin resistance by reducing PMEPA1 mRNA stability through m6A regulation of autophagy [75]. In laryngeal squamous cell carcinoma (LSCC), TMA7 deficiency increases autophagy, while IGF2BP3 prolongs TMA7 mRNA’s half-life, stabilizing it and activating the UBA2-PI3K pathway to inhibit autophagy [76]. In addition, m6A modifications play a significant role in regulating necroptosis. As an m6A reader, hnRNPC not only enhances ATF4 activity and synthesis by modulating the m6A modification of ATF4 but also triggers ER stress, leading to apoptosis and necroptosis in thyroid follicular epithelial cells [77]. LPS induces m6A methylation and upregulates METTL3 expression, while Si-METTL3 reverses LPS-induced oxidative stress, necroptosis, and NF-κB activation, reducing inflammatory factor expression [78]. Interestingly, METTL3 can also inhibit necroptosis. For example, METTL3 mediates ATF3 m6A modification, promoting ATF3 binding to regulate the downstream gene cFLIP, thus inhibiting RIPK1-induced apoptosis and necroptosis in intestinal epithelial cells [79]. Moreover, METTL3 is involved in necroptosis in CRC, although the specific mechanism remains unclear [80]. These findings highlight the interplay between autophagy, necroptosis, and m6A regulators, as well as the functional differences of these regulators under various physiological and pathological conditions (Figure 3).

In summary, the relationship between m6A modifications and PCD is complex and tightly regulated. This interaction plays a crucial role in organismal development, tissue homeostasis, and disease progression. Gaining a comprehensive understanding of this interplay holds great potential for uncovering new therapeutic avenues for a variety of related diseases.

## 4. M6A and Cardiovascular Diseases

The cardiovascular system is a highly intricate network comprising diverse cell types, including cardiomyocytes (CMs), endothelial cells (ECs), and smooth muscle cells (SMCs), which collectively play an indispensable role in sustaining the growth, development, and repair of the heart and blood vessels. The function of m6A alteration in the cardiovascular system has steadily come to light in recent years.

### 4.1. M6A and Cardiomyocytes

**Figure 4:**
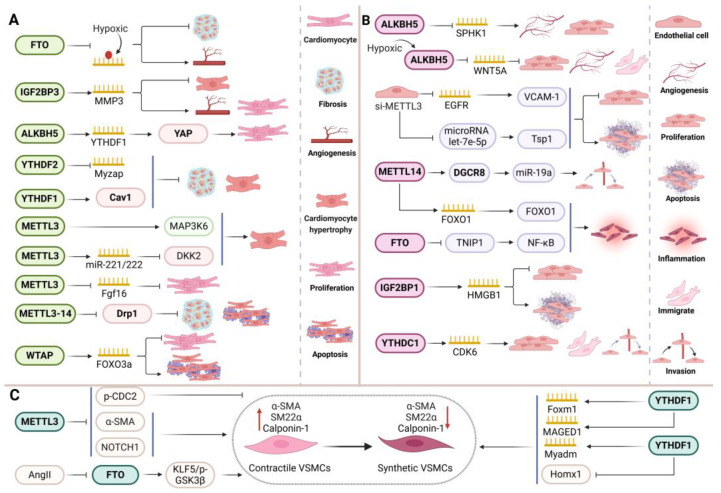
**Figure 4**. The mechanism by which m6A regulates myocardial cells (A), endothelial cells (B), and smooth muscle cells (C) in the cardiovascular system.

M6A mRNA methylation modification plays a critical role in maintaining cardiac homeostasis, function, and response to stress by regulating CMs proliferation, hypertrophy, and fibrosis. FTO is a key regulator of m6A in myocardial cells, and its deficiency leads to reduced ejection fraction and impaired cardiac function [81]. Under hypoxic stress, FTO expression decreases in primary rat CMs; however, overexpression of FTO reverses the hypoxia-induced abnormal increase in m6A, reduces fibrosis, and promotes angiogenesis [82]. IGF2BP3 also protects CMs from stress damage, is upregulated after myocardial injury, improves cardiac function, inhibits acute inflammation and myocardial hypertrophy, and promotes cell proliferation and angiogenesis. Mechanistically, IGF2BP3 may stabilize matrix metalloproteinase 3 (MMP3) mRNA by interacting with m6A methylation through its KH3 and KH4 domains or promote cardiac regeneration independently of MMP3 [83]. As anticipated, silencing ALKBH5 diminishes the cardiomyocyte count and downregulates the proliferation-related gene CTNND1. Conversely, overexpressing exogenous ALKBH5 mitigates myocardial infarction damage by encouraging CMs to re-enter the cell cycle. Further investigation has revealed that ALKBH5 boosts the stability of YTHDF1 mRNA, facilitates efficient YAP translation, and augments cardiomyocyte proliferation [84]. Additionally, the absence of YTHDF1/2 can result in myocardial hypertrophy, fibrosis, and cardiac dysfunction. Specifically, YTHDF2 directly regulates the m6A modification of Myzap, triggering its degradation, whereas YTHDF1 enhances the translation efficiency of the cardiomyocyte membrane raft protein Caveolin1 (Cav1) mRNA, further modulating downstream ERK signaling [85,86]. In contrast, METTL3 promotes myocardial cell hypertrophy [87]. Compared to the control group, METTL3 overexpression elevated the levels of MAP3K6, MAP4K5, and MAPK14 in myocardial cells, leading to a significant increase in cell size. However, knocking out METTL3 resulted in a reduction in myocardial cell cross-sectional area, effectively blocking hypertrophy [88]. Further studies suggest that METTL3 facilitates the maturation of miR-221/222 by increasing m6A modification on pri-miR-221/222, which in turn inhibits DKK2 and activates the Wnt/β-catenin/c-Myc pathway, ultimately inducing myocardial hypertrophy [89]. Additionally, METTL3 negatively regulates Fgf16 mRNA levels through the m6A-YTHDF2 mechanism, limiting CMs proliferation and cardiac regeneration [90]. Interestingly, inhibition of METTL3-14 was found to reduce the translation efficiency of Drp1 mRNA and Drp1 protein levels via 5′UTR m6A modification, thereby decreasing mitochondrial fragmentation and myocardial cell death [91]. In H9C2 rat CMs stimulated by hypoxia/reoxygenation (H/R) and I/R rat models, WTAP is increased similarly to METTL3’s action. This elevation activates the m6A modification site in the 3′UTR of FOXO3a mRNA through YTHDF1, hence reducing cardiomyocyte proliferation [92]. Furthermore, by influencing the degree of m6A modification on certain mRNA transcripts, METTL14, RBM15, ALKBH5, and YTHDF2 control the change of myocardial cell destiny; however, the precise mode of action is yet unknown [93]. Overall, all investigations demonstrate that modifications to the m6A transcriptome play a pivotal role in regulating cardiac cell function (Figure 4A). 

### 4.2. M6A and Vascular Endothelial Cells

M6A modification regulates the expression of specific proteins and genes in vascular ECs (VECs), involving processes such as proliferation and apoptosis, and affects RNA stability and splicing, regulating biological functions. Chronic ischemia damages endothelial cell tube formation, while ALKBH5 contributes to angiogenesis during acute ischemia. ALKBH5 silencing inhibits AKT/eNOS phosphorylation, increases the mRNA m6A level of endothelial angiogenesis regulator SPHK1, leading to protein degradation, but does not affect VEGF-A, indicating that ALKBH5 affects angiogenesis independently of VEGF-A [94]. However, another study suggests that ALKBH5 is a negative regulator of angiogenesis that is only upregulated during hypoxia, reducing WNT5A stability through m6A and impairing the proliferation, migration, and angiogenesis of cardiac microvascular endothelial cells [95]. METTL3 is also a key regulatory factor for EC activation and AS generation. EC-METTL3 deficiency in carotid artery ligation mice leads to increased EGFR expression and exacerbates atherosclerosis. On the contrary, overexpression of METTL3 reverses this pathological process and stabilizes EGFR mRNA through m6A modification [96]. As expected, METTL3 depletion also reduces m6A RNA levels in human umbilical vein ECs (HUVECs) and human cardiac microvascular ECs (HCMECs), leading to reduced cell proliferation and increased apoptosis. This process is achieved by METTL3 by reducing mature angiogenesis microRNA let-7e-5p and miR-17-92 clusters and increasing thrombospondin 1 (Tsp1) [97]. Conversely, METTL14 stimulates the expression of FOXO1 by bolstering the m6A modification and translation of its mRNA, which subsequently amplifies endothelial inflammation and elevates the expression of adhesion molecules such as VCAM-1 and ICAM-1, thereby fostering the progression of atherosclerosis [98]. Furthermore, heightened expression of METTL14 facilitates the interaction between methylated RNA and DGCR8, enhances the m6A modification of pri-miR-19a and its maturation into miR-19a, ultimately accelerating the proliferation and invasion of AS VECs [99]. MeRIP-Seq analysis reveals that FTO inhibits TNIP1 mRNA expression by demethylating m6A modifications on TNIP1, subsequently activating the NF-κB pathway and exacerbating endothelial damage [100]. In parallel, studies have demonstrated that HG levels upregulate the expression of IGF2BP1 in VECs, leading to increased cell apoptosis and decreased proliferation. Mechanistically, IGF2BP1 stabilizes m6A-modified RNA by interacting with HMGB1 mRNA, hinting at its potential as a therapeutic target for vascular complications in diabetes [101]. YTHDC1, functioning as an m6A reader protein, facilitates HG-induced proliferation, migration, invasion, and angiogenesis in retinal VECs by upregulating CDK6 methylation, ultimately exacerbating ECs damage [102]. However, downregulation of YTHDC1 was detected in hypoxic pulmonary artery ECs (PAECs), and YTHDC1 inhibited the proliferation of hypoxic pulmonary artery endothelial cells (HPAECs) and pulmonary vascular remodeling [103]. It can be seen that the function of YTHDC1 varies among different tissues and may also depend on its expression level and function under specific physiological or pathological conditions. In summary, m6A modification can synergistically interact with various genes encoding growth factors, receptors, signal transduction molecules, etc., and significantly affect the proliferation, migration, and formation of vascular networks in endothelial cells (Figure 4B).

### 4.3. M6A and Vascular Smooth Muscle Cells

In vascular SMCs (VSMCs), m6A modification holds a pivotal role by modulating the expression of specific genes, thereby influencing crucial biological behaviors including proliferation, migration, and phenotype transformation. When subjected to stress or pathological conditions, highly differentiated VSMCs undergo dedifferentiation, a process characterized by reduced expression of smooth muscle contraction markers (α-SMA, SM-MHC, calpain, calprotectin, and sm22-α), alongside heightened levels of cell growth signals, fibrosis, inflammation-related molecules, and MMPs [104]. METTL3 inhibits the proliferation, migration, and phenotypic transition of human aortic SMCs (HASMCs) by promoting autophagosome formation. Overexpression of METTL3 arrests the G2/M cell cycle transition while enhancing the expression of contractile markers such as α-SMA, SM22α, and calmodulin-1. Additionally, it downregulates the expression of MMP2, MMP7, and MMP9 [105]. These findings suggest that elevated METTL3 levels and the activation of autophagy may delay neointimal hyperplasia. Another study supports this by showing that silencing METTL3 reduces the expression of VSMCs-specific markers [106]. However, contrasting findings indicate that METTL3 overexpression increases m6A modification at the NOTCH1 3′UTR, leading to the suppression of NOTCH1 and a reduction in smooth muscle markers like α-SMA, SM22α, and calmodulin, which ultimately promotes HASMCs proliferation [107]. The influence of varying environmental conditions and the involvement of other related molecules may significantly affect the distribution, function, and expression of METTL3. The specific mechanisms underlying these discrepancies remain unclear and warrant further investigation. FTO influences VSMCs via m6A demethylation, fostering VSMC phenotype transformation. Specifically, FTO diminishes m6A modification of KLF5 mRNA, leading to elevated KLF5 and p-GSK3β levels, which subsequently stimulates cell proliferation and migration [108]. In pulmonary artery SMCs (PASMCs) exposed to hypoxia and PH, both YTHDF1 and m6A levels are markedly upregulated. YTHDF1 functions on two fronts: it enhances forkhead box M1 (Foxm1) translation efficiency in an m6A-dependent manner, bolstering PASMCs proliferation, and it recognizes and augments MAGED1 translation, contributing to PASMCs proliferation, phenotype transition, and PH development [109,110]. Moreover, YTHDF2 plays a role in the pathophysiological processes of PH. Notably, mice with bone marrow-specific YTHDF2 knockout exhibit alleviation of PH and PASMCs proliferation, potentially through YTHDF2-mediated m6A-dependent degradation of heme oxygenase 1 (Homx1) mRNA [111]. Additionally, YTHDF2 overexpression in SMCs stabilizes MyADM mRNA in an m6A-dependent manner, promoting PASMCs proliferation and right ventricular hypertrophy. Thus, inhibiting YTHDF2 or MyADM may represent a novel therapeutic approach for this condition [112]. Collectively, these studies underscore the pivotal role of m6A modification in governing VSMCs phenotype transition and development by modulating VSMCs proliferation, migration, and inflammatory capacity. Consequently, m6A modification influences vascular homeostasis and disease progression. These discoveries offer a fresh perspective on the regulation of VSMC function and hold promise for identifying novel therapeutic targets for CVD (Figure 4C).

## 5. M6A and Programmed Cell Death in Cardiovascular Disease

The impact of CVD on myocardial cells, endothelial cells, and smooth muscle cells is interconnected, collectively influencing the cardiovascular system to induce alterations in vascular structure, functional deterioration, and organ damage. Despite this, the underlying cellular and molecular mechanisms remain elusive. Recently, m6A modification has emerged as a key regulator of PCD in CVD, thereby modulating the pathological and physiological processes of these conditions.

### 5.1. Myocardial Ischemia-Reperfusion

**Figure 5:**
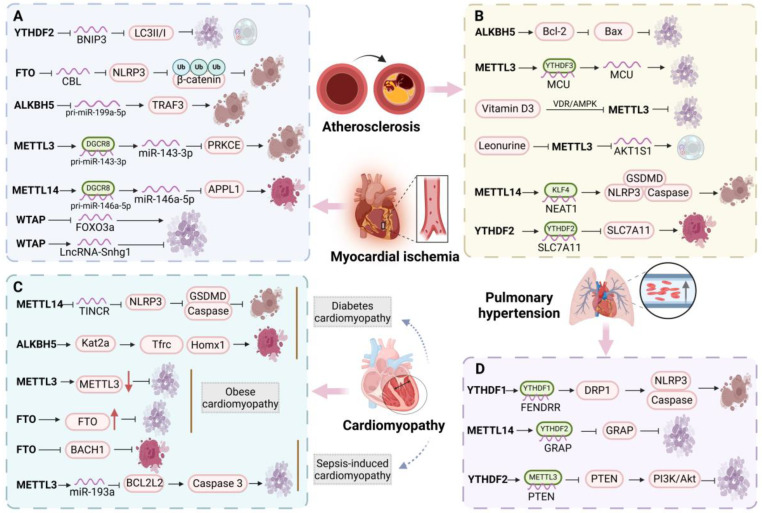
**Figure 5**. The relationship between m6A and programmed cell death in myocardial ischemia (A), atherosclerosis (B), cardiomyopathy (C), and pulmonary hypertension (D).

I/R injury is a complex cardiovascular pathological event closely related to PCD [113]. M6A affects the tolerance of myocardial cells to injury by regulating the stability or translation efficiency of mRNA associated with PCD (apoptosis, pyroptosis, ferroptosis, and necroptosis). H/R treatment decreased the survival rate of myocardial cells, yet upregulation of YTHDF2 can counteract this adverse effect. By modifying m6A, it inhibits BNIP3 and thereby mitigates cell apoptosis and autophagy resulting from myocardial ischemia-reperfusion injury (MIRI) [114]. FTO also regulates CMs pyroptosis. Overexpression of FTO can block NLRP3 inflammasome-mediated pyroptosis induced by oxygen glucose deprivation/reoxygenation (OGD/R), reduce mRNA m6A modification of CBL, decrease its stability, and thus inhibit CBL-mediated β-catenin ubiquitination degradation, ultimately protecting the myocardium from I/R injury [115]. In contrast to the above effects, ALKBH5 inhibits the maturation and expression of pri-miR-199a-5p by reducing its m6A methylation, and then regulates the high expression of downstream TNF receptor-related factor 3 (TRAF3), thereby promoting pyroptosis and aggravating MIRI progress [116]. Similarly, METTL3 and METTL14 are upregulated in MIRI and OGD/R models. By raising m6A levels and promoting DGCR8 binding to pri-miR-143-3p, METTL3 upregulates miR-143-3p, blocking PRKCE transcription and aggravating cardiomyocyte pyroptosis in MIRI damage [117]. By preventing the Akt/mTOR signaling pathway from being activated, METTL14 controls myocardial necrosis and apoptosis while enhancing oxidative stress and the inflammatory response [118]. Apart from apoptosis, METTL14 also improves pri-miR-146a-5p precursor recognition and processing by DGCR8 through m6A methylation levels, miR-146a-5p production, APPL1 transcription inhibition, and H/R-induced ferroptosis [119]. WTAP triggered the m6A modification site on the 3′UTR of FOXO3a mRNA through YTHDF1, enhancing the stability of FOXO3a mRNA and promoting apoptosis and inflammatory cytokine infiltration in rat CMs, which contributes to H/R-induced I/R injury [92]. However, recent reports have indicated that WTAP promotes m6A modification of LncRNA-Snhg1, enhancing its protective effect on the myocardium, while also targeting the miR-361-5p/OPA1 axis to regulate CMs apoptosis and mitochondrial ROS generation, thereby improving the MIRI process [120]. In summary, these studies demonstrate the important role of PCD and m6A in the pathophysiological process of myocardial ischemia, providing new directions for further research (Figure 5A). 

### 5.2. Atherosclerosis

The earliest significant change in AS is VEC dysfunction. ECs exhibit various forms of PCD, including apoptosis, necroptosis, ferroptosis, and autophagy, often accompanied by inflammatory cell infiltration [121]. M6A modification plays a role in atherosclerosis progression by influencing endothelial cell PCD mechanisms. ALKBH5 may prevent the degradation of Bcl2 mRNA through demethylation in HUVECs, leading to a downregulation of Gadd45, Bax, and p21, thereby inhibiting TNF-α-induced apoptosis in HUVECs. Furthermore, ALKBH5 might also inhibit miR-7 maturation to enhance Bcl2 levels, though this requires further validation [122]. Infection of VECs with human cytomegalovirus (HCMV) leads to a significant increase in m6A modification, primarily driven by the upregulation of METTL3. METTL3 methylates the MCU on three m6A residues in the 3′UTR, enhancing the binding of YTHDF3 to the methylated MCU mRNA and facilitating its translation, which exacerbates HCMV-induced endothelial cell apoptosis [123]. However, the pro-apoptotic effects of METTL3 can be countered by vitamin D3 and the alkaloid compound leonurine. The former reduces cell apoptosis by inhibiting METTL3 via the VDR/AMPK pathway, while the latter decreases AKT1S1 stability through METTL3 inhibition, thus activating autophagy and reducing lipid accumulation [124]. In addition to apoptosis and autophagy, METTL14 also promotes pyroptosis in ECs. It binds to the m6A site of NEAT1, enhancing its expression through YTHDC1. This interaction facilitates NEAT1’s binding to KLF4, leading to the upregulation of NLRP3, caspase-1 p20, GSDMD, IL-1β, and IL-18 protein levels, which subsequently promotes the development of AS [125]. Moreover, YTHDF2 exacerbates ferroptosis in ECs within the context of AS. The overexpression of YTHDF2 inhibits the proliferation of HUVECs and accelerates ferroptosis. Mechanistically, YTHDF2 binds to SLC7A11 mRNA via m6A modification, promoting its degradation and reducing mRNA stability [126]. In summary, m6A modification and programmed cell death play a crucial role in the pathophysiology of AS. Investigating novel m6A-related programmed cell death pathways in AS may offer promising avenues for future research (Figure 5B).

### 5.3. Pulmonary Arterial Hypertension

PH is a severe cardiovascular condition characterized by elevated pressure in the pulmonary arteries and remodeling of the pulmonary vasculature. This remodeling process initially involves dysfunction and apoptosis of PAECs, followed by increased proliferation of PASMCs [127]. In PH patients and animal models, the expression of m6A-modified mRNA is altered, influencing cell proliferation, migration, and apoptosis, and ultimately affecting pulmonary vascular remodeling. Previous studies have established that the lncRNA FENDRR is implicated in the pathogenesis of hypoxic PH. The protein YTHDC1 targets the m6A modification site on FENDRR, resulting in decreased stability and significant downregulation of FENDRR in hypoxic PAECs. This downregulation subsequently increases the methylation of the DRP1 promoter via RNA-DNA triple-stranded formation, promoting DRP1 transcription and elevating levels of pyroptosis-related markers (NLRP3, pro-caspase-1, and IL-1β), thereby facilitating hypoxia-induced pyroptosis in PAECs [128]. In hypoxic conditions, the levels of METTL14 and YTHDF2 significantly rise. Notably, METTL14 employs YTHDF2 as a mediator to enhance the m6A modification of GRAP mRNA, leading to the degradation of GRAP and further advancing the pathological processes associated with PH [129]. Similarly, other studies have indicated that YTHDF2 promotes the degradation of PTEN by recognizing m6A-modified PTEN mRNA mediated by METTL3. The subsequent reduction in PTEN activates the PI3K/Akt signaling pathway, resulting in excessive proliferation of PASMCs. However, downregulation of METTL3 can reverse this process [130]. It is well established that the PI3K/Akt signaling pathway holds a significant correlation with cell death, and it can be hypothesized that METTL3 may affect the death process of PASMCs [131]. These observations suggest that the axes involving YTHDC1/FENDR/DRP1, METTL14/YTHDF2/GRAP, and METTL3/YTHDF2/PTEN play crucial roles in the hypoxia-induced phenotypic modulation of PASMCs and PCD. This provides a novel insight into the molecular mechanisms underlying the abnormal proliferation of PASMCs (Figure 5C).

### 5.4. Cardiomyopathy

A condition known as cardiomyopathy is defined as having defective cardiac shape and function. Primary cardiomyopathy includes diabetic cardiomyopathy (DCM) and obese cardiomyopathy, whereas secondary cardiomyopathy includes sepsis-induced cardiomyopathy (SICM) [132]. The pathogenic process of cardiomyopathy can be controlled by controlling the activity of m6A methyltransferase or demethylase, which in turn can control the processes of pyroptosis, ferroptosis, apoptosis, and metabolism of cardiac cells. Significant downregulation of METTL14 was seen in the cardiac tissue of DCM rats. The expression of factors linked to apoptosis (NLRP3, caspase-1, and GSDMD-N) is inhibited by the overexpression of METTL14. Mechanistically, YTHDF2-regulated RNA degradation caused by METTL14-mediated m6A alteration reduces TINCR expression by decreasing NLRP3 stability. This, in turn, results in NLRP3 downregulation and impedes the development of pyroptosis and DCM [133]. Apart from pyroptosis, m6A controls ferroptosis in DCM. By lowering m6A methylation on Kat2a mRNA, ALKBH5 encourages Kat2a upregulation. Afterwards, Kat2a promoted ferroptosis by enriching the H3K27ac and H3K9ac promoter areas, which in turn increased the expression of ferroptosis genes (Tfrc and Homx1) [134]. Downregulating METTL3 and upregulating FTO in obese cardiomyopathy mitigates lipid accumulation and cardiomyocyte death brought on by high-fat diets [135]. Furthermore, FTO exhibits a protective function in SICM by modulating m6A modification of BACH1 to decrease its levels, thereby inhibiting ferroptosis [136]. Conversely, METTL3 facilitates the maturation of pri-miR-193a into miR-193a through enhanced m6A modification. The elevated miR-193a subsequently binds to and downregulates BCL2L2, activating the caspase-3 apoptotic pathway. This leads to cardiomyocyte apoptosis, inflammatory responses, and exacerbation of SICM [137]. These findings underscore the intricate interplay between m6A and PCD, offering fresh perspectives and potential therapeutic avenues for cardiomyopathy and related cardiac injury treatment strategies (Figure 5D).

### 5.5. DOX-Induced Cardiotoxicity

**Figure 6:**
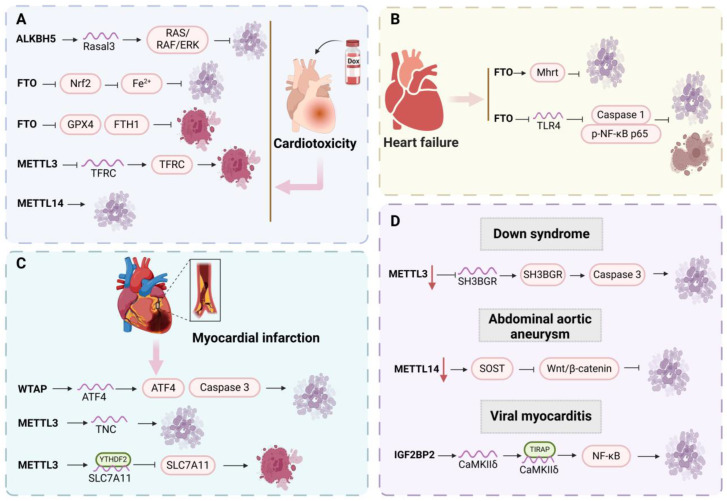
**Figure 6**. The relationship between m6A and programmed cell death in DOX induced cardiotoxicity (A), heart failure(B), myocardial infarction(C), and other cardiovascular diseases(D).

The toxic effects of DOX can impair myocardial cells, progressively leading to pathological cardiomyopathy [138]. In this progression, diverse cell death mechanisms, including apoptosis, ferroptosis, and copper death, play crucial roles, with m6A modification closely associated with this intricate toxic process. Recent studies have noted increased mortality in ALKBH5-deficient mice, whereas overexpression of ALKBH5 modulates Rasal3 mRNA stability in an m6A-dependent manner, activating the RAS/RAF/ERK pathway to inhibit CMs apoptosis and alleviate doxorubicin-induced cardiomyopathy (DIC) [139]. Following FTO knockout, there is an elevation of myocardial fibrosis genes, oxidative stress factors, and iron overload, exacerbating DOX-induced apoptosis, ROS accumulation, and myocardial fibrosis in mice [140]. Additionally, FTO inhibits ferroptosis by downregulating GPX4, FTH1, HO-1, xCT, and TFR expressions. Specifically, it ameliorates DIC in mice and H9C2 cells through HuR-dependent m6A demethylation of P53 and P21/Nrf2 mRNA, activating these protective factors [141]. Conversely, METTL3 knockout significantly improves heart function decline caused by DOX, effectively mitigating iron accumulation and cardiomyocyte death, whereas METTL3 overexpression exhibits the opposite effect. Mechanistic analysis revealed that METTL3 modifies TFRC mRNA (a key gene regulating iron uptake) with m6A, enhancing its stability [142]. Moreover, METTL14 knockout prevents CMs apoptosis by reducing total RNA m6A methylation, demonstrating a protective effect on the myocardium by alleviating TUNEL staining and mitochondrial ROS accumulation [140]. Notably, in sepsis-induced cardiac toxicity, the expression of cuproptosis-related genes (CRGs) such as CD274, CP, VEGFA, COX11, CCL8, etc., is significantly altered, with a notable correlation between differential regulatory genes of CRGs and the expression levels of m6A methylation genes [143]. Although the specific mechanisms involved remain incompletely understood, these findings provide insights and directions for exploring the intricate mechanisms linking m6A, cuproptosis, and cardiac toxicity. In summary, the regulation of cardiac toxicity by m6A through PCD is a complex and pivotal process, with the detailed molecular mechanisms meriting further investigation (Figure 6A). 

### 5.6. Heart Failure

HF, as a cardiovascular syndrome, is the main cause of decreased cardiac function, hospitalization, and death [144]. M6A affects this process by influencing cell apoptosis, fibrosis, and gene expression changes. In the HF mouse model, downregulation of FTO and Mhrt expression was observed, while an increase in m6A total levels and Mhrt m6A levels was observed. Further exploration revealed that FTO can upregulate the expression of Mhrt in CMs under H/R treatment and reduce its m6A modification, thereby effectively inhibiting cardiomyocyte apoptosis [145]. In addition, FTO also plays a protective role in Dox-induced HF. As expected, FTO reduced m6A methylation of TLR4 mRNA through YTHDF1, inhibited the expression of TLR4, p-NF-κB p65, p-IκB-α, LDH, and MDA, and prevented caspase-1-dependent inflammatory cell apoptosis, thereby alleviating cell apoptosis, inflammation, pyroptosis, and oxidative stress [146]. The above indicates a close relationship between m6A methylation modification and heart failure. Through in-depth research on the relationship between m6A modification and heart failure-related gene expression, myocardial cell death, proliferation, and repair processes, new ideas and targets can be provided for the treatment of heart failure (Figure 6B).

### 5.7. Myocardial Infarction

MI represents a severe cardiovascular condition that poses a significant threat to life, potentially causing an abrupt reduction or cessation of blood flow within the coronary arteries, ultimately leading to myocardial cell damage [147]. The process of m6A methylation plays a pivotal role in regulating myocardial cell apoptosis, ferroptosis, ER stress, and fibrosis by modulating the expression of specific mRNAs. Initially, WTAP impacts the expression of ATF4 mRNA through its regulation of m6A methylation. The ER stress and apoptosis pathways mediated by WTAP are contingent upon ATF4. Both in vitro and in vivo studies have validated the function of WTAP in myocardial I/R injury, where it facilitates ER stress and cell apoptosis via m6A modification of ATF4 mRNA. This modification leads to an upregulation in the expression amount of cleaved PARP, cleaved caspase-3, ATF4, and CHOP, thereby exacerbating MI [148]. Secondly, METTL3 elevates the m6A level of TNC mRNA and enhances its stability, contributing to cardiac fibrosis and myocardial cell apoptosis post-MI. Inhibiting METTL3 can mitigate this effect [149]. METTL3 also has the ability to bind with SLC7A11, promoting its m6A methylation. The m6A reader YTHDF2 recognizes the methylated SLC7A11 and reduces its stability. In contrast, silencing METTL3 inhibits OGD/R-induced ferroptosis by blocking YTHDF2’s recognition of SLC7A11 m6A methylation [150]. This suggests that METTL3 is a crucial factor in promoting apoptosis and fibrosis induced by myocardial hypoxia, and its regulation of ferroptosis may represent a potential therapeutic target for MI. The above results reveal that PCD is involved in the progression of m6A modification-regulated MI; therefore, it is important to further explore the molecular mechanisms involved (Figure 6C).

### 5.8. Others

In addition to the aforementioned CVD, m6A also affects the progression of Down syndrome (DS), abdominal aortic aneurysm, and viral myocarditis by regulating cell apoptosis and inflammation. DS is a genetic disease caused by additional duplication of trisomy 21, which affects heart growth and development [151]. Researchers have detected a significant decrease in METTL3 expression in DS heart tissue, while there was no significant change in the expression of METTL14 and FTO. Transcriptomic analysis revealed that SH3BGR was the most significantly increased gene in DS heart. Interestingly, METTL3 deficiency enhances the expression of SH3BGR by inhibiting the decay of SH3BGR mRNA while significantly increasing the apoptosis process of striated muscle cells in mice, accompanied by a large amount of caspase-3 expression in cardiac tissue [152]. It can be seen that METTL3 may partially play a role in the process of embryonic cardiac apoptosis by regulating SH3BGR expression. In abdominal aortic aneurysm, inhibition of METTL14/YTHDC1-mediated m6A modification can increase mRNA stability, leading to increased expression of osteocalcin (SOST) in diseased VSMCs and human aortic SMCs, followed by inactivation of the Wnt/β-catenin signaling pathway, preventing VSMCs phenotype transition, apoptosis, and vascular inflammation [153]. In addition, in viral myocarditis mice and primary CMs intervened with Coxsackievirus B3 (CVB3), CaMKII δ was upregulated, while knockout inhibited apoptosis and improved cell survival and proliferation. This process is attributed to IGF2BP2 stabilizing CaMKII δ, enabling it to interact with toll/interleukin-1 receptor domain containing adapter protein (TIRAP) to activate the NF-κB pathway, inhibit cell survival and proliferation, promote apoptosis, and thus affect VMCs outcomes (Figure 6D).

## 6. The Impact of Exercise on Cardiovascular Disease by Regulating m6A and Programmed Cell Death

It is well accepted that exercise, being a potent non-pharmacological intervention, improves cardiovascular health [154,155]. Through diverse mechanisms, exercise is capable of modulating m6A modification and PCD processes, thereby exerting a positive influence on CVD. Studies have demonstrated that key mediators involved in exercise-induced physiological cardiac hypertrophy facilitate cardiomyocyte growth, bolster cardiomyocyte resistance to apoptosis, and curb ECs pyroptosis [156]. Notably, METTL14 and YTHDF2 play pivotal roles in regulating this exercise-induced hypertrophy. Swimming training, for instance, results in a reduction in overall mRNA m6A levels in the heart, accompanied by the downregulation of METTL14, a crucial m6A enzyme. This downregulation activates Akt-S473 by regulating the m6A modification of PHLPP2 mRNA, which in turn promotes cardiomyocyte growth, inhibits apoptosis, and ultimately suppresses pathological cardiac remodeling [157]. Furthermore, METTL14 significantly impacts endothelial cell pyroptosis and atherosclerosis through exercise. It typically binds to the m6A site of NEAT1 and promotes NEAT1 expression via YTHDC1 recognition, thereby inducing endothelial pyroptosis. However, further research has revealed that NEAT1 activates NLRP3 protein transcription by binding to KLF4, exacerbating endothelial cell pyroptosis, but this process can be mitigated by exercise, leading to improvements in atherosclerosis [125]. In the case of YTHDF2, it is downregulated during exercise-induced physiological myocardial hypertrophy. Exercise affects the dynamic balance of myocardial cells by reducing protein lactylation and inhibiting YTHDF2 and its downstream target, Ras GTPase activating protein binding protein 1 (G3BP1), thereby inhibiting OGD/R-induced apoptosis, alleviating myocardial pathological remodeling, and improving myocardial MIRI [158]. Similarly, exercise training also reduces WTAP levels in rats, inhibiting the installation of m6A modifications on downstream FOXO3a messengers, which in turn decreases cardiomyocyte apoptosis and the expression of inflammatory cytokines [92]. This suggests that exercise alleviates H/R-induced myocardial cell (H9C2) and I/R injury in rats by altering the WTAP/YTHDF1/m6A/FOXO3a axis. Moreover, treadmill training in mice with HF with preserved ejection fraction (HFpEF) results in higher total m6A levels, downregulation of FTO protein levels, inhibition of myocardial cell apoptosis, myocardial fibrosis, and myocardial hypertrophy. However, overexpression of FTO offsets the beneficial effects of exercise training on these mice, indicating that FTO is a key target for improving HF through exercise training [159].

According to the aforementioned information, exercise training influences the PCD process and controls the expression of particular genes via m6A, which reduces the incidence and progression of cardiovascular illnesses. This offers exercise therapy a fresh theoretical foundation. Future studies will investigate the precise processes behind exercise-induced epigenetic alterations and PCD, as well as the variations in these pathways among various cardiovascular disease types.

## 7. Conclusions and Prospect

M6A is the most prevalent internal RNA modification in eukaryotes, playing a multifaceted role in numerous biological processes [160,161]. PCD is an active cellular process that helps maintain internal environmental stability [162]. Both m6A methylation and PCD are involved in the pathophysiology of various human diseases, influencing their onset and progression [50,71]. In recent years, advancements in high-throughput sequencing technology and bioinformatics have led to the identification of a growing number of m6A modification sites. These sites are widely distributed across the genomes of different organisms and are strongly associated with various biological processes, such as cell differentiation, development, brain function, and disease pathogenesis [163,164]. Notably, in the context of cell death, m6A modification has been shown to regulate multiple forms of PCD, including apoptosis, pyroptosis, ferroptosis, autophagy, and necroptosis.

M6A plays a crucial role in regulating PCD, influencing various biological processes and contributing to CVDs such as I/R injury, AS, PHA, cardiomyopathy, DIC, HF, and MI. M6A-related proteins regulate PCD by affecting the processing, splicing, stability, translation efficiency, and interactions of specific target mRNAs with other RNA molecules [165]. Studies have shown that certain M6A-related proteins are abnormally expressed during PCD and contribute to the development of CVD by regulating the homeostasis of CMs, VECs, and VSMCs. However, the precise regulatory mechanisms linking m6A modification to PCD remain unclear. Current research primarily focuses on how m6A “writers”, “erasers”, and “readers” influence apoptosis, pyroptosis, and ferroptosis, the three main types of PCD. There is still limited understanding of how M6A-related proteins affect other forms of PCD, necessitating further investigation.

CVDs encompass a range of conditions affecting the heart and blood vessels, and they remain a leading cause of death and disability worldwide [166]. Currently, there are no fully effective treatments or medications available for many of these conditions. Recent studies suggest that abnormal expression of m6A-related proteins in CVD may lead to disruptions in PCD, contributing to both the pathophysiology and progression of these diseases. These proteins may serve as potential therapeutic targets. Therefore, research into m6A modifications and their associated proteins is expected to provide new therapeutic strategies for CVD. By modulating m6A levels or the expression of related proteins, it may be possible to influence PCD processes, reduce cardiac damage, and improve heart function.

Overall, m6A plays a crucial role in regulating PCD in CVD and represents a promising therapeutic target. As research continues, more molecular mechanisms linking PCD and m6A will likely emerge. Developing targeted drugs targeting m6A methyltransferase or demethylase can regulate the level of m6A modification, thereby affecting the biological behavior of myocardial and vascular cells and achieving the goal of treating CVD. Develop more effective lifestyle intervention strategies (such as diet, exercise, etc.) based on the regulatory mechanism of m6A modification to improve the prevention and treatment effectiveness of CVD. This not only helps us better understand the pathogenesis of CVD, but may also open up new interesting avenues for cardiovascular research.
